# Therapeutic Antibodies for the Treatment of Respiratory Tract Infections—Current Overview and Perspectives

**DOI:** 10.3390/vaccines9020151

**Published:** 2021-02-13

**Authors:** Alexie Mayor, Adélaïde Chesnay, Guillaume Desoubeaux, David Ternant, Nathalie Heuzé-Vourc’h, Thomas Sécher

**Affiliations:** 1 INSERM, Centre d’Etude des Pathologies Respiratoires, U1100, F-37032 Tours, France; alexie.mayor@univ-tours.fr (A.M.); adelaide.chesnay@univ-tours.fr (A.C.); guillaume.desoubeaux@univ-tours.fr (G.D.); nathalie.vourch@univ-tours.fr (N.H.-V.); 2 Faculty of Medecine, Université de Tours, F-37032 Tours, France; david.ternant@univ-tours.fr; 3 CHRU de Tours, Parasitologie—Mycologie—Médecine Tropicale, F-37044 Tours, France; 4 CHRU de Tours, Pharmacologie Médicale, Pôle Biologie Médicale, F-37044 Tours, France; 5 CHRU de Tours, Centre Pilote de suivi Biologique des Traitements par Anticorps (CePiBAc), F-37044 Tours, France; 6 EA 7501 GICC—PATCH, F-37032 Tours, France

**Keywords:** therapeutic antibodies, respiratory tract infection, pharmacodynamics (PD), pharmacokinetics (PK), clinical development

## Abstract

Respiratorytract infections (RTIs) are frequent and life-threatening diseases, accounting for several millions of deaths worldwide. RTIs implicate microorganisms, including viruses (influenza virus, coronavirus, respiratory syncytial virus (RSV)), bacteria (*Pseudomonas aeruginosa*, *Streptococcus pneumoniae*, *Staphylococcus aureus* and *Bacillus anthracis*) and fungi (*Pneumocystis* spp., *Aspergillus* spp. and very occasionally *Candida* spp.). The emergence of new pathogens, like the coronavirus SARS-CoV-2, and the substantial increase in drug resistance have highlighted the critical necessity to develop novel anti-infective molecules. In this context, antibodies (Abs) are becoming increasingly important in respiratory medicine and may fulfill the unmet medical needs of RTIs. However, development of Abs for treating infectious diseases is less advanced than for cancer and inflammatory diseases. Currently, only three Abs have been marketed for RTIs, namely, against pulmonary anthrax and RSV infection, while several clinical and preclinical studies are in progress. This article gives an overview of the advances in the use of Abs for the treatment of RTIs, based on the analysis of clinical studies in this field. It describes the Ab structure, function and pharmacokinetics, and discusses the opportunities offered by the various Ab formats, Ab engineering and co-treatment strategies. Including the most recent literature, it finally highlights the strengths, weaknesses and likely future trends of a novel anti-RTI Ab armamentarium.

## 1. Introduction

Respiratory tract infections (RTI) constitute the third leading cause of morbidity and mortality worldwide, in both children and adults, accounting for approximately 4.25 million deaths *per* year [1,2]. In addition to premature mortality, RTIs have a dramatic impact on society with respect to disability, healthcare costs and loss of productivity. Thus, the prevention and control of RTIs constitute a major goal of public health.

RTIs refer to a range of infections confined to the upper respiratory tract (rhinitis, sinusitis, pharyngitis or tracheitis) and/or lower respiratory tract (mainly bronchitis and pneumonia), implicating microorganisms, including viruses, bacteria and fungi. According to the World Health Organization (WHO), pneumonia is more lethal in children than the combined mortality from measles, malaria and acquired immune deficiency syndrome (AIDS) [3,4]. 

After more than sixty years, characterized by the use and misuse of broad-spectrum antibiotics, resistance to antibiotics and some antivirals has become a real threat, while thedeficit of drugs with novel modes of action to implement the conventional armamentarium represents a critical concern. The attrition of existing therapeutic options and emergence of new viruses has complicated the management of RTIs, increasing pressure on healthcare systems and motivated the development of new therapeutic strategies for RTIs. Among these, antibodies (Abs), and antibody-based molecules, are now considered as a viable option against emerging viral pathogens and antibiotic-resistant bacteria [5,6,7,8,9].

The pioneer work of Von Behring regarding a diphtheria anti-toxin serum therapy paved the way for the current use of Abs in infectious diseases. Later on, Cecil and Larsen were among the first to conduct a case–control group study to test the efficacy of polyclonal antibody against *Streptococcus pneumoniae*. Although the success of this study was modest, it allowed to concretely consider Ab therapy in RTIs [10]. Abs present potential advantages compared to other antimicrobials. First, they provide a therapeutic option when vaccines or conventional drugs are neither available nor efficacious, due to compromised immune system or intrinsic resistance. Secondly, Abs usually target highly conserved, specific antigens and are associated with limited development of drug resistance. Finally, in immunocompetent patients, they may provide a prophylactic setting, acting as vaccine-like molecules through the promotion of long-term anti-microbial specific immune responses [11].

Despite extensive investments in Ab discovery and manufacturing, there are currently only three therapeutic Abs approved by the health authorities for fighting against RTIs (Table 1), and fewer than twenty in clinical development. This highlights the complexity of anti-infective Ab development, especially in the field of infectious diseases, which needs to overcome scientific, regulatory and even commercial barriers before being accessible to patients. 

In this review, we described the pharmacodynamics (PD) and pharmacokinetic (PK) features of all anti-infectious monoclonal Abs against RTIs that either have been approved by regulatory agencies or are in clinical development for RTIs, and outlined their strengths and limitations. Considering the relevance of Abs to benefit respiratory infections due to emerging pathogens [12], we also highlighted the Abs developed to fight the SARS-CoV-2 pandemic. 

## 2. Methods

We undertook a search on Clinical.Trials.gov regarding trials for recombinant monoclonal antibodies in respiratory infectious diseases referenced up to January 1, 2021. The term used in different combinations were as follows: “therapeutic antibodies”, “monoclonal antibodies” and pathogen-specific terms (including: *Bacillus anthracis*, *Klebsiella pneumoniae*, *Mycoplasma pneumoniae*, *Streptococcus pyogenes*, *Streptococcus pneumoniae*, Group A *Streptococcus*, *Chlamydia pneumoniae*, *Chlamydia trachomatis*, *Chlamydia psittaci*, *Moraxella catarrhalis*, *Haemophilus influenza*, *Bordetella pertussis*, *Coxiella burnetii*, *Legionella pneumophila*, *Mycobacterium tuberculosis*, *Corynebacterium diphtheriae*, *Neisseria gonorrhoeae*, *Neisseria meningitis*, *Francisellatularensis*, *Xanthomonas pseudomallei*, *Yersinia pestis*, *Escherichia coli*, *Enterobacter*, *Proteus*, *Aspergillus* spp., *Pneumocystis jirovecii*, *Cryptococcus* spp., *Candida* spp., *Coccidioides immitis*, *Blastomyces dermatitidis*, *Paracoccidioides brasiliensis*, *Paragonimus* spp., *Histoplasma capsulatum*, *Respiratory syncytial virus*, *Influenza A virus*, *Influenza B virus*, *Human papillomavirus*, *Rhinovirus*, *Adenovirus*, SARS-CoV-2 and COVID-19). The results were limited to on-going trials or trials involving entities for which development is still active (survey of the sponsoring company pipeline). They were confirmed on several drug databases (https://www.antibodysociety.org; https://adisinsight.springer.com/;https://www.covid-trials.org/. Our methodology was further documented by specific searches on Pubmed.

## 3. Result

### 3.1. Therapeutic Antibodies—Main Features

Since the development of the hybridoma technology by Köhler and Milstein, in the early 1970s [13]—who earned the Nobel Prize for this work—Ab and Ab-derived molecules have emerged as anti-infective options, even for emerging viral infections [5,14]. Numerous technological advances have been done to improve the efficacy of Abs. From the first-generation murine Ab, innovative approaches have given rise to chimeric, humanized and fully human Abs, which can be sequenced, engineered and bio-produced at a large scale [15,16]. The present review will focus on the immunoglobulin G (IgG) scaffold since most Abs approved or under investigation in RTI are full-length IgG. IgG contain an antigen-binding fragment (Fab) recognizing a targeted antigen with a high specificity and affinity, hindering any side effect on symbiotic microflora. They also comprise a fragment crystallizable (Fc) region, mediating the downstream effectors function through its interaction with immune cells (via the engagement with various Fc receptors) or the complement component C1q. The Fc domain (via the engagement with the neonatal receptor for IgG, FcRn) is also intricately associated with a favorable PK, theoretically conferring to IgG a ≈ 21 day-long half-life (T½) in biological fluids [17]. The mechanisms of action of Ab are multiple and attributable to their Fab region, their Fc region or both. They include direct-killing, neutralization, virulence inhibition, complement deposition, opsonophagocytosis, antibody-dependent cell-mediated cytotoxicity (ADCC) or complement-dependent cytotoxicity (CDC) [14,17]. 

The structural and functional properties of the natural human IgG subclasses—IgG1, IgG2, IgG3 and IgG4—can be used to generate therapeutic Abs [18]. Indeed, subclasses differ with regard to molecular structure, PD and PK profiles. However, most Abs on the market are from the IgG1 subclass, most likely because of the stability of IgG1 during the manufacturing process, the greater potency of the effector functions and serum bioavailability [19]. 

In addition to a multi-faceted PD, the Ab PK is unique, involving specific mechanisms of absorption, catabolism and elimination. The fate of the Ab depends on multiple parameters, thereby their PK displays a large inter-individual variability, which can be detrimental for clinical responses [20]. Understanding the parameters governing the Ab PK better remains critical to enhance the likelihood of an Ab response during RTI.

### 3.2. Therapeutic Antibodies against Virus Diseases

#### 3.2.1. Respiratory Syncytial Virus (RSV)

Human respiratory syncytial virus (RSV) belongs to the *Paramyxoviridae* family. It is an enveloped, single-stranded, negative-sense RNA virus. Its enveloped lipid bilayer contains three membrane proteins: The small hydrophobic protein (SH), the attachment glycoprotein (G) and the fusion protein (F) [21,22]. It is noteworthy that the approved and under evaluation (even discontinued) Abs indicated for RSV infection are all directed against the F protein. Indeed, the F protein has an essential role in RSV pathophysiology: The virus binds to host cell using the G protein, and thereafter uses the F protein to fuse with the host cell membranes. As compared to G protein, F protein exhibits a more conserved sequence among the RSV strains and is more immunogenic [23] (Table 2). It is noteworthy that F proteins exist in two forms—the prefusion (pre-F) and post-fusion (post-F) conformations—which may impact antibody binding [23].

HA (hemagglutinin), PD1 (programmed death 1) and PDL1 (programmed death-ligand 1) are responsible for infections, usually limited to the upper airways, including low-febrile rhino-pharyngitis; but, it can spread deeper into the respiratory tract, leading to bronchiolitis or pneumonia. Sensitivity to RSV infection is person-dependent, but mostly involve critical cases at the extremes ages of life and especially in young children during the first two years of life; indeed, a large majority of children contracts RSV and up to 40% developed a lower respiratory tract infection [24,25]. Despite the health care burden associated with the RSV, there is no curative antiviral drug (except the controversial ribavirin) or vaccine. The main treatment strategy relies on supportive care to relief nasal congestion, fever, dehydration or hypoxia.

Additionally, for supportive care, palivizumab (Synagis^®^) was approved in 1998 for prophylaxis of respiratory disease caused by RSV in children at high risk. This humanized IgG1 Ab is the only approved drug targeting specifically the RSV, more precisely, an epitope present both in the pre-F and post-F conformations. Efficacy was demonstrated among children with bronchopulmonary dysplasia, with congenital heart disease or premature infants (Impact-RSV Trial). 

Besides, two additional anti-RSV Abs are currently in clinical development: MEDI-8897 (nirsevimab) is a fully human IgG1 Ab developed by AstraZeneca for the prevention of RSV infection [26] (Table 2). Compared to palivizumab, MEDI-8897 is 9-fold more effective in reducing viral load in cotton rats. This improved efficacy could be partly explained by the specific targeting of the pre-F conformation. Indeed, it has been observed that the vast majority of potent neutralizing antibodies isolated from the serum of convalescent patients exclusively recognize the pre-F form, which has led to the development of pre-F-specific antibodies as novel therapeutic approaches for RSV infection. It presents also an extended T½ (63–73 days in child) consecutive to the introduction of an YTE modification in the Fc fragment. In a Phase 1b/2a (NCT02290340) trial, anti-drug antibodies (ADA) were detected, but were not associated with adverse effects nor a reduction in blood Ab concentration, for up to 151 days. Based on a Phase 3 clinical trial (NCT03979313), MEDI-8897 is expected to improve the prevention against RSV infection in late premature and healthy full-term children. MK-1654 is a fully human Ab-targeting RSV F protein, developed by Merck. It also contains an YTE mutation to increase its serum T½ [27]. An interim report from a Phase 1 study (NCT03524118) helped to characterize a PK model (developed for adults), able to predict PK in children and supports further development in children [27]. 

#### 3.2.2. Human Papillomavirus (HPV)

HPV, a well-known driver of genital tract lesions and cervical cancer, may also be responsible for recurrent respiratory papillomatosis (RRP). RRP is characterized by a papilloma growing in the respiratory tract, and can lead to voice changes, pulmonary lesions, airway compromise and fatal distal airway obstruction [28]. In 2018, Ahn et al. stained and scored by automated cell count 39 formalin-fixed, paraffin-embedded RPP for CD4, CD8, FoxP3 and PD-1 [29]. They showed that most RRP specimens demonstrated PD-1 (programmed cell death-1) T-lymphocyte infiltration and PD-L1 (programmed death-ligand 1) expression on both the papilloma and infiltrating immune cells, suggesting that this checkpoint pathway may contribute to local immunosuppression in RRP. Two clinical trials are in progress to determine whether re-purposed immunotherapies targeting the PD-L1 and PD-1 checkpoint pathway, originally developed to treat cancer patients, can be used to activate the immune system against HPV-infected cells (Table 2). Avelumab is a human IgG1 that binds to PD-L1 and blocks the interaction between PD-L1 and its receptors, PD-1 and B7.1. This leads to the suppression of the inhibitory effects of PD-L1 on cytotoxic CD8+ T cells, thus restoring the anti-tumor responses of the T lymphocytes. Avelumab also induces direct lysis of tumor cells by natural killer cells via ADCC [30]. A Phase 2 study (NCT02859454) was conducted to investigate the clinical activity and safety of avelumab in patients with RRP in a two-stage design, with initial enrollment of 12 patients and expansion to 37 patients, if one or more complete response(s) is/are observed in the initial group. The initial enrollment was achieved in 2019 [31]. All patients with laryngeal RRP experienced improvement in disease burden after treatment with avelumab, while no response was observed in patients suffering from pulmonary RRP. Some of the responders developed HPV-specific reactivity in papilloma-infiltrating T-cells that correlated with a reduced viral load. Pembrolizumab, a humanized IgG4k directed against PD-1, prevents the interaction of PD-1 with its two known ligands PDL-1 and PDL-2 present on tumor cells [32]. In a Phase 2 study (NCT02632344), the investigators are looking at whether pembrolizumab may restore the natural ability of the immune system to recognize and eliminate HPV-infected cells.

#### 3.2.3. Influenza Virus

Influenza viruses are negative-sense, single-strand RNA viruses belonging to *Orthomyxoviridae* family. There are three types of human influenza viruses—A, B and C—defined according to the expression of nucleoprotein (NP) and matrix (M) protein. Type A and, to a lesser extent, type B are the most frequently encountered in the human population. Their envelope displays two main surface proteins called hemagglutinin (HA) and neuraminidase (NA). HA is implicated in virus entry in host cells via binding to sialic acid, preferentially in the upper airways. NA is involved in the release of the newly produced virus particles. Influenza A viruses are classified into subtypes based on HA (H1–16) and NA (N1–9) antigenic differences. Influenza viruses are responsible for seasonal influenza, which is typically limited to the upper airways and generally leads to mild symptoms, including fever, sneezing, sore throat, coughing, headaches, myalgia and asthenia. However, influenza can eventually be severe when associated with secondary bacterial infection, culminating in devastating pneumonia, especially in children, elderly and chronically disabled persons. Influenza causes 290,000 to 650,000 deaths *per* year worldwide, with a mortality rate estimated to be 0.2% [33]. For preventive approaches, inactivated, live attenuated or recombinant HA vaccines are available [34]. However, small mutations in HA and NA, referred to as antigenic drift, frequently occur during each epidemic season and lead to influenza evasion from immune responses. Consequently, the vaccine composition is adjusted and renewed every year. Beside these small changes, major genetic recombination may occur, giving rise to viruses with entirely novel HA or NA, responsible for pandemic flus such as influenza A/H1N1 in 2009. Thus, curative treatments are also necessary. Two main strategies have been considered: (i) to limit the spread of the virus with an NA inhibitor (oseltamivir, peramivir, zanamivir and laninamivir) or M2 ion-channel inhibitor (amantadine and rimantadine);and (ii) to block the viral replication with polymerase acidic (PA) endonuclease inhibitors (baloxavirmarboxil) [35].

However, the emergence of resistant viruses increases the need for new treatments and the interest for anti-influenza Abs. All Abs currently in clinical development target HA, considering its essential role on the virus lifecycle. It should also be noted that all Abs are directed against influenza A viruses (Table 2). VIS-410 is a fully human IgG1Ab (Visterra) binding to the stem region of HA, to neutralize the virus. VIS-410 appeared to be well tolerated up to a dose of 50 mg/kg (NCT02045472) [36]. In a Phase 2 placebo-controlled trial, VIS410 demonstrated a significant anti-influenza activity (NCT02989194, NCT02468115) [37,38]. The results of a study comparing VIS410 in association with oseltamivir to oseltamivir alone (NCT03040141) showed that addition of VIS410 induced a decrease inmortality and faster virus clearance [39]. Celltrion has developed CT-P27, containing two monoclonal Abs directed against influenza HA. In vivo and in vitro studies demonstrated its efficacy against several influenza A subtypes and a Phase 1 trial (KCT000161) showed positive safety results. A Phase 2 trial (NCT02071914) is ongoing. VIR-2482 (VIR Biotechnology) is an engineered Ab targeting a conserved region of HA. It is intended for flu prophylaxis, since it has a longT½. A Phase 1/Phase 2 (NCT04033406) study was launched in 2019 to assess its safety, pharmacokinetics, immunogenicity and efficacy.

#### 3.2.4. The Severe Acute Respiratory Syndrome Coronavirus 2 (SARS-CoV-2) Pandemic

**Figure 1:**
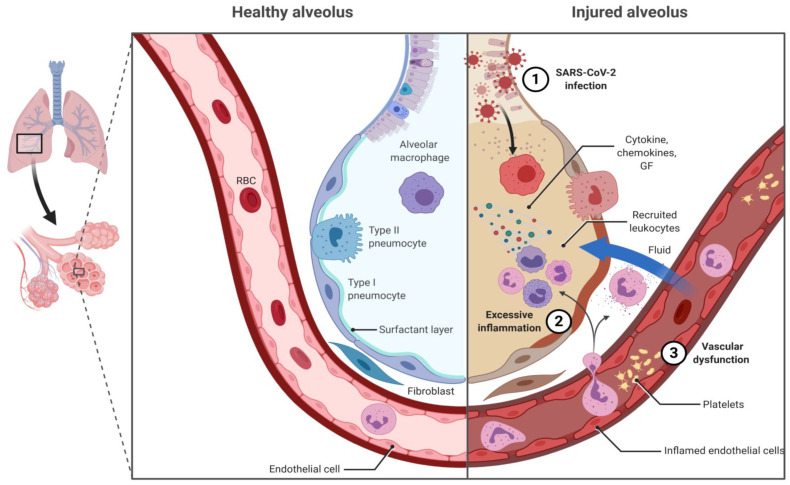
This figure compares the alveolar immune responses in healthy and injured lung after SARS-CoV-2 infection. (**1**) Coronavirus infects lung cells and encounter alveolar macrophages. (**2**) Alveolar macrophages identify the virus and initiate innate and acquire immune responses. Cytokines, chemokines or growth factors will attract additional immune responses, which will amplify and perpetuate the inflammation, damaging lung alveoli and endothelium. (**3**) Endothelium/platelet activation and increased vascular permeability will ultimately provoke vascular dysfunction, allowing virus dissemination and boost leukocytes infiltration.

SARS-CoV-2 is a *Betacoronavirus*, like Severe Acute Respiratory Syndrome Coronavirus (SARS-CoV) and Middle East Respiratory Syndrome Coronavirus (MERS-CoV), which are positive-sense, single-stranded RNA viruses. It consists of four major structural proteins: spike glycoproteins (S), forming spikes that hoop from the virus surface; membrane glycoproteins (M); envelope proteins (E), which are transmembrane; and hemagglutinin-esterase dimer protein (HE), containing acetyl-esterase activity; it has a 30 Kb RNA genome. Viral entry in host cells is achieved by exploiting the link with membrane-bound angiotensin converting enzyme II (ACE2) protein, which is expressed in different pulmonary cell types (type II alveolar cells, upper airways cells) and across the human body (e.g., endothelial cells, kidney or ileum epithelial cells). However, macrophages may also be infected by the SARS-CoV-2 [40]. This probably participates in the extrapulmonary complications associated with SARS-CoV-2. However, pulmonary infection caused by SARS-CoV-2 is the most life-threatening, resulting in some patients in an acute respiratory distress syndrome (ARDS), characterized by an impaired innate immunity, inadequate adaptive immune response, uncontrolled virus- and inflammation-induced tissue damage and vascular permeability (Figure 1) [41]. Much of our understanding of SARS-CoV-2 immunity comes from the incredible amount of studies that has been done during the pandemic and comparison with SARS-CoV/MERS-CoV. Nonetheless, the pathogenesis of COVID-19 remains to be fully elucidated. Briefly, binding of S glycoprotein to ACE2 initiates SARS-CoV-2 entry into host cells. Fusion of the viral and cellular membranes mainly uses the serine protease TMPRSS2 (transmembrane serine protease 2) and endosomal cysteine proteases (cathepsin B and L). Once SARS-CoV-2 RNA is liberated in the host cell, it is translated and replicated—like an mRNA—using the host cell machinery. After packaging, the neo-virus is released extracellularly by budding, exocytosis and host cell death. SARS-CoV-2 is a cytopathic virus causing direct cell death during viral replication, resulting in tissue damage and an increased inflammatory response. SARS-CoV-2 replication has been shown throughout the respiratory tract.

The immune response to the virus infection usually starts at the cellular level, and plays a critical role in COVID-19 pathogenesis [42]. On one hand, SARS-CoV-2 will induce antiviral immune responses—both innate and adaptive immune responses—with protective features to control viral infection. It may start in the endosomal compartment of infected host cells, through signaling of pattern-recognition receptors, such as Toll-like receptor 3, which will elicit production of inflammatory cytokines and, in turn, activate the immune cells. Innate immune cells, such as dendritic cells, macrophages and neutrophils, can be activated by cytokines and damage-associated molecular patterns (DAMPs), and initiate adaptive immunity responses. On the other hand, SARS-CoV-2 may elicit pathogenic immune responses. After virus entry into cells, membrane ACE2 is downregulated, leading to an imbalance in the renin–angiotensin pathway, thereby promoting angiotensin II-induced vascular permeability and pneumonia with vascular injury. Moreover, activation of Type 1 Angiotensin II Receptor directly upregulates NF-kB and ADAM17, ultimately leading to uncontrolled production of TNF-α and IL-6 [43]. During COVID-19 pathogenesis, activated alveolar macrophages and lung epithelial cells are the major producer of these mediators [44], which, in turn, will at a systemic level mediate emergency myelopoiesis and locally increase the expression of cell adhesion molecules and VEGF [41]. The resulting increase in lung vascular permeability will allow viral dissemination as well as leukocyte infiltration, perpetuating local inflammation, leading to ARDS (Figure 1).

Over the past few months, many Abs have been developed to face the SARS-CoV-2 pandemic [41,43,44,45]. We can distinguish two categories of anti-COVID-19 Abs. The first category of anti-COVID-19 Abs is intended to reduce the symptoms and comprise mostly re-purposed Abs, targeting excessive inflammation. The second category includes Abs or Ab-based therapeutics directly targeting SARS-CoV2 (Table 3).

Regarding the first category, several clinical trials are evaluating the potential use of antibodies targeting pro-inflammatory cytokines or their receptors. Among them, two are already approved for the treatment of COVID-19 in Russia: olokizumab and levilimab. Olokizumab, a humanized anti-IL6 IgG4 (R-Pharm International), was approved in May 2020, for patients suffering from severe COVID-19 respiratory distress. Levilimab (Biocad) is a human IgG1 that binds to soluble and membrane-bound IL-6 receptor and was also approved in Russia, in June 2020. In addition, re-purposed Abs, already approved as treatments for other diseases, are the subject of strong interest with numerous clinical trials. Sarilumab (Sanofi/Regeneron) is a human IgG1 targeting the interleukin-6 receptor. It is approved for rheumatoid arthritis in adults and currently tested in Phase 3 for hospitalized patients with COVID-19, alone or in association to tocilizumab. Tocilizumab (Hoffmann-LaRoche) is a humanized IgG1 also targeting IL-6R, marketed for several indications and is currently tested in 42 clinical trials in COVID-19 patients. First results in Europe are promising, showing an improvement of clinical outcome in patients with moderate or severe COVID-19 pneumonia [46]. Siltuximab (EUSA Pharma, Hempstead, UK and BeiGene Ltd., Beijing, China) is a chimeric IgG1 targeting IL-6, indicated in patients with multicentric Castleman’s disease and evaluated in Phase 3 in COVID-19 (NCT04330638). Siltuximab already showed promising results in patients with COVID-19 respiratory failure, when used as compassionate treatment [47]. Emapalumab (Swedish Orphan Biovitrum) is a human IgG1 raised against interferon gamma, approved for treatment of primary hemophagocytic lymphohistiocytosis, and evaluated in a Phase 2/3 in patients with COVID-19 (NCT04324021). Considering the involvement of the dysregulated complement cascade in severe COVID-19 complications, ravulizumab (Alexion Pharmaceuticals), a humanized IgG2/4 directed against C5, marketed for treatment of paroxysmal nocturnal hemoglobinuria, was evaluated in Phase 3 (NCT04369469). 

The second category of anti-COVID-19 Abs, corresponding to more than half (including discovery and preclinical stages), directly targets SARS-CoV2. The majority are raised against S proteins to prevent binding of the virus on host cells. Among the Ab-based therapeutics in development, there are polyclonal Abs, fusion proteins, single domain Abs and DARPin, but the majority are monoclonal Abs. Among them, eight molecules already reached clinical trials: some are proposed as an antibody cocktail (REGN-COV2, AZD7442, AMD03820), some were purified from a convalescent patient (LY3819253, CT-P59, JS016, AZD7442, HFB30132A) and most of them are fully-human monoclonal Abs. Several of those Abs have already reached phase 3 (LY-CoV555/LY3819253, REGN-COV2, SCTA01, TY027, VIR-7831, AZD7442, LY3832479, CT-P59) and two of them (LY3819253, REGN-COV2) received an Emergency Use Authorization (EUA) [48]. However, the Abs most likely to be authorized in the next months are Abs from plasma of recovering COVID-19 patients, which may benefit to hospitalized COVID-19 patients until other biotherapeutics are available. It is noteworthy that an antibody is evaluated by the inhalation route as well as by intravenous injection (BI767551 DZIF-10c, Boehringer Ingelheim). A matter of concern for the development of vaccines or therapeutic Abs against SARS-CoV2 is antibody-dependent enhancement of viral entry (ADE), enhancing viral entry into cells expressing the Fc receptor for immunoglobulins. Although ADE has not been demonstrated yet for SARS-CoV-2, neutralizing Abs without an effector function may be appropriate to prevent ADE. 

### 3.3. Therapeutic Abs against Bacterial Diseases

With the exception of *Haemophilus influenza* type B mediating severe epiglottitis and laryngotracheitis and *Streptococcus pyogenes* causing pharyngitis, and for which no antibodies are under development, bacteria are mostly associated with lower respiratory tract infections (mainly pneumonia) [49]. The most common etiologic agent of pneumonia is *Streptococcus pneumoniae*, which has a greater incidence in children and the elderly [50]. Other frequent causes of bacterial pneumonia, often with a multidrug-resistant phenotype, include *Pseudomonas aeruginosa*, *Staphylococcus aureus*, *Haemophilus influenza* and also bacteria grouped as atypical pneumonia (*Mycoplasma pneumoniae*, *Chlamydia* spp., *Legionella* spp. or *Coxiella burnetii*) [50].

Pneumonia is characterized by an infection of the lung parenchyma. It occurs when the immune system is unable to eliminate a pathogen from the alveolar compartment [51]. The impairment of host immunity is either due to a pathogen’s active mechanisms (toxin or virulence factors production) or to comorbidities (immunodeficiency, impaired mucociliary clearance, airway obstruction). The pathogen gives rise to a protracted local inflammation, which will alter lung mechanics and physiology and perpetuate damage to the lung parenchyma, accelerating systemic inflammation and worsening prognosis [52]. In the next sections, we will present and discuss the most recent advances in Abs targeting bacterial pathogens involved in RTI (Table 4).

#### 3.3.1. Bacillus anthracis

*Bacillus anthracis* is a spore-forming, rod-shaped, Gram-positive, facultative anaerobic bacterium, responsible for a zoonotic disease called anthrax. Humans can develop anthrax through exposure to infected animals or their products. The clinical manifestation of human anthrax varies accordingly to the mode of entry of the bacterium, with pulmonary anthrax (inhalation of spores) being the most fatal form. Apart from its animal origin, anthrax is considered to be a major bioterrorism threat. After inhalation, clinical symptoms develop usually within a week and resemble those of influenza or community-acquired pneumonia [53]. Without any treatment, pulmonary anthrax mortality ranges between 50 and 80% and coincides with the production of two main virulence factors: A capsule, which precludes the phagocytosis by host immune cells, and the tripartite anthrax toxin. Anthrax toxin is composed of a protective antigen (PA), the edema factor (EF) and the lethal factor (LF) [54]. PA allows the binding, molecular re-arrangement and endocytosis of EF and LF in host cells, ultimately causing swelling and cell death. The essential role of PA in the pathophysiology of anthrax has been extensively documented and makes this protein an attractive therapeutic target [55]. Bioterrorism anthrax attacks in the USA in 2001 provoked massive investments in medical countermeasures, which resulted in the marketing of raxibacumab (ABthrax^®^—2012) and obiltoxaximab (Anthim^®^—2016), two Abs that neutralize PA [56,57]. Raxibacumab is a human IgG_1_, whereas obiltoxaximab is a chimeric IgG_1_. A Phase 3 study in 333 human volunteers (NCT00639678) showed higher tissue concentrations of raxibacumab than those that were shown effective in animals [57]. In monkeys, obiltoxaximab allowed 100% survival if administered one to three days before anthrax exposure, and 83 to 100% survival, if given 18 to 24 h after challenge [57]. The two Abs are indicated in adult and pediatric patients for the treatment of pulmonary anthrax in combination with standard-of-care antibacterial drugs. It is noteworthy that there is not any data related to pediatric populations, and the dose level and regimen were extrapolated from a population-based PK approach. In the clinical practice, raxibacumab is indicated for the treatment of anthrax pulmonary disease in adults at the intravenous dose of 40 mg/kg, in children at 40 to 80 mg/kg in combination with adequate conventional antibiotics, and in prophylaxis if alternatives are not feasible. Obiltoxaximab is used in the same indications at the dosages of 16 mg/kg in adults and 16 to 32 mg/kg in children. However, premedication with diphenhydramine is recommended prior to obiltoxaximab administration, because it was reported to induce hypersensitivity reactions and anaphylaxis in healthy subjects [57], when it was given intravenously at 16 mg/kg. Due to the concern about adverse reactions, a Phase 4 trial has recently started (NCT03088111) to assess the clinical benefit of the use of obiltoxaximab given intravenously. Recently, very good tolerance of obiltoxaximab in a randomized open-label Phase 1 trial in healthy volunteers (NCT01952444) by intramuscular route (up to 24 mg/kg) was reported [58].

#### 3.3.2. Staphylococcus aureus

*Staphylococcus aureus* is an opportunistic Gram-positive pathogen with a significant worldwide healthcare burden. A substantial part of the human population is naturally colonized by this bacterium. Nonetheless, *S. aureus* has the ability to change from a commensal phenotype to an actual pathogen involved in various clinical manifestations, of which the most serious is pneumonia, especially in hospitalized patients [59]. The recent increasing prevalence of methicillin-resistant *Staphylococcus aureus* (MRSA) strains has become a global health problem attributable to a high mortality rate [60], complicating anti-microbial therapy. In contrast to most bacterial pathogens, *S. aureus* has the ability to secrete a large array of cytotoxins [61]. In particular, alpha-hemolysin (Hla or AT) is a key virulence factor. Once bound to its receptor (ADAM10) on the host cell surface, Hla oligomerizes to form heptameric transmembrane pores, which may lyse epithelial, endothelial and immune cells. To overcome host defenses, *S. aureus* also produces five leukocidins (SF-PV, LukED, LukGH, HlgAB and HlgCB), which target myeloid and lymphoid cells [62].

Two Abs are currently under investigation and aim at preventing or limiting *S. aureus*-related disease through the neutralization of AT. MEDI-4893 (suvratoxumab, by AstraZeneca) has been shown to be protective in multiple animal models of pneumonia [63,64,65]. It is a high affinity IgG1 with an extended T½ (YTE mutated). During a Phase 2 trial, it was shown to slightly improve lung function of patients with *S. aureus* infection and was associated with low adverse events, overall encouraging AstraZeneca to continue its clinical development [66]. AR-301 (salvecin), developed by Aridis Pharmaceuticals, was designed as an adjunctive therapy to standard-of-care anti-*S. aureus* antibiotics. Even if there was no preclinical data published, the results of a Phase 2 trial were recently presented and showed a significant reduction in time under mechanical ventilation in Ab- plus antibiotics-treated patients as compared to the placebo, with good tolerance [67]. These promising results prompted a Fast Track designation by the FDA (Food and Drug Agency), an orphan-drug designation by the EMA (European Medicines Agency) and the design of a Phase 3 trial (NCT03816956), which is currently enrolling patients.

#### 3.3.3. Pseudomonas aeruginosa

*Pseudomonas aeruginosa* (Pa) is another important nosocomial pathogen responsible for devastating acute pneumonia, in immunocompromised, ventilated patients or cystic fibrosis patients with immunologically-quiet chronic colonization. Pa versatility is related to the genetic flexibility of its large genome [68]. Many bacterial surface antigens have been considered for the development of therapeutic Abs, with disappointing results. For instance, the most clinically advanced Ab is AR-101 (aerumab), a human IgM binding to lipopolysaccharide (LPS) serotype O11. LPS is an important component of the *P. aeruginosa* outer membrane and has been historically used for serologic typing before genomic methods were available. In a Phase 2 trial, AR-101 given in combination with standard-of-care anti-*P. aeruginosa* antibiotics proved to be well tolerated and decrease the time to pneumonia resolution as compared to the placebo [69]. Additional bacterial antigens have been considered for the development of Abs in *P. aeruginosa* strains associated with chronic colonization. Aridis Pharmaceuticals developed AR-105 (aerucin), a human IgG1, directed against *P. aeruginosa* biofilm—a complex extracellular polymeric matrix that is produced by the bacteria to increase its resistance to the host’s immune cells and against antimicrobial therapy, thereby favoring its long-term survival in the host. AR-105 targets alginate, one of the three main exopolysaccharides (with PsI, Pel) composing the biofilm [70]. It successfully completed a Phase 1 clinical trial in 16 healthy volunteers, with a good tolerance up to 20 mg/kg. The FDA has granted AR-105 with a fast-track designation and Aridis Pharmaceuticals initiated a Phase 2 trial in the second quarter of 2017. Results were expected to be reported in the third quarter of 2019, but there is not any information yet.

### 3.4. Therapeutic Abs against Fungal Diseases

Few therapeutic Ab have been developed against fungal diseases. Allergic bronchopulmonary aspergillosis (ABPA) is the main focus of current research interests (Table 5). ABPA usually develops in patients with chronic respiratory diseases, particularly in asthmatics or in pediatric cystic fibrosis patients. Triggered by hypersensitivity immune reactions to *Aspergillus* antigens in a context of chronic *Aspergillus* tracheobronchial colonization, ABPA has an inflammatory pathology, characterized in particular by augmented titers in specific anti-*Aspergillus* IgE [71]. The main objective of ABPA therapy is to control episodes of acute inflammation and limit the progression of lung injury. Two Abs, benralizumab and dupilumab, are under clinical trials and target components of the host immune responses. Benralizumab is a humanized IgG1 that binds to the IL-5Rα subunit, on eosinophils and basophils. The absence of fucose on the Fc fragment of benralizumab results in a high affinity for FcγRIII receptors present on the surface of immune effector cells, such as natural killer cells. This causes apoptosis of eosinophils and basophils through ADCC. In 2019, a Japanese team reported the case of a patient with ABPA successfully treated with benralizumab [72]. A Phase 4 (NCT04108962) open-label study is now evaluating the effects of benralizumab in the treatment of severe asthma in patients with allergic bronchopulmonary aspergillosis. Dupilumab is a humanized IgG4 that binds to the IL-4Rα subunit, thereby inhibiting IL-4 and IL-13 signaling and decreasing IgE production. A Phase 3 study (NCT04442269) is evaluating the efficacy and safety of dupilumab, in participants with ABPA, in particular the annualized rate of exacerbations. 

### 3.5. Pharmacokinetics of Anti-Rti Abs

#### 3.5.1. Administration Route

Historically, Abs were mainly administered intravenously (IV). However, an increasing number of molecules have been developed for the subcutaneous (SC) or intramuscular (IM) routes, the latter being used for palivizumab and obiltoxaximab. Absorption following the SC or IM route occurs by convective transport via lymphatic system, leading to a time of peak blood concentration ranging from two to 10 days [56]. The distribution of an Ab is limited by their hydrophilicity and high molecular mass: its central and steady-state volumes of distribution are, respectively, 3–5 and 5–15 L. Elimination of Abs occurs via three main mechanisms: (i) endogenous catabolism; (ii) target-mediated elimination; and (iii) elimination mediated by ADA.

#### 3.5.2. Endogenous (Non-Specific) Catabolism

After cellular uptake, IgGs bind to neonatal Fc receptor (FcRn) in acidic pH conditions. FcRnensures their protection from lysosomal degradation and recycling into the bloodstream, which explainsan IgG’s long elimination (T½). Accordingly, most anti-infectious Abs—as they are IgGs—have a T½ of approximately 20 days. The increasing knowledge in Ab molecular biology has granted the emergence of Ab engineering with specific mutations that modulate the Ab’s properties. In particular, mutations in the Fc portion were specifically designed to increase their affinity towards FcRn. This allowed for MEDI-4893 to have dose-proportional concentrations and to sustain serum exposure above the target level 30 days after infusion, which may provide a longer duration of disease prevention [73]. The administration of Abs in children has been a challenge, especially anti-RSV Abs in premature infants. In general, Ab elimination seems to increase with young age [20]. The reasons remain unclear, even if a possible explanation is a decreased FcRn expression in infants. This may explain the lower T½ of nirsevimabin infants than in adults [26,74].

#### 3.5.3. Target-Mediated (Specific) Elimination

Abs bind to their antigen target with a high affinity; the Ab–target complex is then eliminated by the immune system. Thus, Abs are drained by antigen targets. The target-mediated elimination rate is correlated to the target amount and turnover, and the elimination rate of the Ab–target complexes. Target-mediated elimination results in nonlinear elimination decay, which further complicates the design of optimal dosing [75].

Target-mediated elimination is often reported in dose-ranging studies, where an increase of T½ with dose is observed, as for obiltoxaximab [58]. At the tested doses, almost all anti-infectious Abs presented linear elimination decay. However, the majority of PK studies were achieved in healthy subjects, who present null or low amounts of targets and therefore no target-mediated elimination. Extrapolating the PK findings in healthy subjects to define the dose and regimen in RTI patients may not be appropriate, since target-mediated elimination of Abs may be increased in infected patients, which may ultimately lead to underexposure and treatment failure.

#### 3.5.4. Elimination Mediated by Anti-Drug Antibody

When investigated in Ab PK studies, the presence of ADA is mostly associated with increased Ab clearance, and thus decreased exposure that altogether can lead to loss of efficacy. The presence of ADA is observed in approximately 2–20% of patients, variable with antibody and underlying disease [20], primarily with palivizumab [76] and nirsevimab [26]. 

#### 3.5.5. Pharmacokinetic Variability, Concentration-Response Relationship and Optimal Dosing

The PK of anti-infectious Ab is variable among patients and may be influenced by several factors, including age, the presence of ADA and the amount of antigen targets (viruses, bacteria or target cells). This variability leads to a complex PK behavior for some Abs, which complicates the design of an optimal dose in therapeutics. In humans, a drug’s PK is quantified using either non-compartmental methods or population compartmental modeling [77]. The latter one has been more and more used, because it allows sound quantification of PK, concentration–effect relationship variability and the design of an optimal dosing regimen. This approach is further needed in case of complex PK due to the influence of antigen burden. Up to date, PK results using this approach were reported only for palivizumab [76]. Overall, the PK of anti-infectious Abs needs further investigations; new clinical studies are needed to soundly quantify the dose-concentration–response relationship and to design optimal dosing strategies for anti-infectious Ab treatments.

## 4. Discussion and Conclusions

Antibiotic resistance has become epidemic with projections estimating mortality from bacterial infections to be around 10 million/year in 2050, surpassing combined deaths from cancer and heart diseases [78]. In particular, the ESKAPE pathogens, comprising four bacteria associated with RTIs (*Staphylococcus aureus*, *Klebisella pneumoniae*, *Acinetobacter baumannii* and *Pseudomonas aeruginosa*), represent a serious healthcare issue, requiring innovative therapeutic strategies. In this context, therapeutic Abs are a promising alternative to antibiotics for infectious diseases and they have already proven successful [14].

Since 2018, with the exception of the anti-SARS-CoV-2 Ab, the pipeline of anti-RTI Abs in clinical development has undergone a significant contraction: nine Abs have been discontinued and no novel Ab has entered into clinical evaluation [14]. This highlights that even if being a thriving focus of research, the development of anti-infectious Abs remains challenging. First, the selection of the antigenic target continues to be disputed. Indeed, many Abs have been designed to bind to epitopes that are not conserved or display strain heterogeneity (e.g., lipopolysaccharides (LPS), exopolysaccharides), questioning the clinical relevance of targeting a unique pathogenic antigen. This strategy is even more arguable for viral infections, considering the rapid evolution and selection of escape mutants, especially for emerging viruses [5]. In addition, extrapolated data from animal models are fraught with conjectures in proper target selection. For example, the development of an anti-*S. aureus* Ab has been hindered with the focus made on surface structure targeting (capsule, lipoteichoic acid) and the aim of opsonophagocytic killing, while neglecting the development of anti-toxin Abs. This was based on a misinterpretation of the toxins’ importance in a mouse model, which are nearly insensitive to leukocidins [63,79]. In this context, refined preclinical models allowing a better understanding of the pathogenesis of lung infections and accelerated diagnostic procedures may be fundamental to improve the success of anti-RTI Abs.

Novel technologies—comprising multivalent antibodies, molecules derived from antibodies or novel formats towards antibody–drug conjugate (ADC) or antibody–antibiotic conjugate (AAC) [80]—may offer alternatives to overcome the antigenic variability of pathogens and resistance to standard treatments. Antibody mixtures, e.g., cocktails targeting different microbial pathogenic mechanisms, or Abs combined to standard-of-care antibiotics and immunomodulators, may help dealing with multidrug-resistant strains (MDR), while avoiding the appearance of cross-resistance and the disruption of the normal microflora of patients. Engineering strategies may also improve the Ab’s PK and/or their effectors functions. 

Another limitation of anti-RTI Ab development is related to their cost and their business environment. Besides the costs associated with the preclinical development of any drug, Ab production is expensive, evaluated to be several tens of thousands of dollars per g of material [81]. Business models and potential markets depend on the pathogen and may be more limited in RTI than inflammatory diseases or cancer. Moreover, development of anti-RTI Abs is hindered by uncertainties about (i) testing preventive or therapeutic settings in patients; (ii) definition of the best-suited population for the intervention; and (iii) the choice of appropriate end-points, especially when evaluating them in acute RTIs [82]. Altogether, these concerns make future economic returns unsure, which may possibly explain the discontinuation of several anti-RTI Ab programs. However, it would be reasonable to put the Ab cost in view of the average expense for the treatment of patients suffering from MDR lung infections, which is estimated to be increasingly high [78,83]. Solutions may come from innovations (i) in Ab production (plant cell-based or DNA plasmid-delivered muscle cell-based), which will improve yields; and (ii) in Ab delivery (considering novel intramuscular or inhalation routes), which will reduce therapeutic dosing while improving efficacy [14,84]—both concurring to limit production costs.

Notably, the last few months has witnessed the fundamental place of Abs to fight emerging pathogens. Overall, 13 clinical trials evaluating Abs against SARS-CoV-2 are ongoing (1). Immunomodulating Abs, such as tocilizumab, already showed promising results, most likely preventing SARS-CoV-2 inflammatory-induced lung damage. This increasing global trend is evidence for a strong dynamic that could place therapeutic Abs as an effective complement to the conventional anti-RTI arsenal. Along with improvements in production and engineering, this should fuel the expansion of an Ab-based anti-RTI armamentarium. However, additional studies are required to decipher more clearly their PK, especially in diseased patients rather than in healthy subjects, and to investigate alternative delivery routes, like inhalation. All these issues represent substantial costs regarding preclinical and development processes, but the recent SARS-CoV-2 pandemic underscored how relevant and critical it is to promote alternative therapeutic strategies and anti-pathogen preparedness.

## Figures and Tables

**Table 1:** Approved therapeutic antibodies for respiratory infections.

Indication	Generic Name	Sponsoring Company	Target	Development Stage
*Respiratory syncytial virus*	Palivizumab (synagis)	AstraZeneca	F-protein	Approved in 1998
*Bacillus anthracis*	Raxibacumab (abthrax)	GSK	PA	Approved in 2013
	Obiltoxaximab (anthim)	Elusys Therapeutics	PA	Approved in 2016

PA, protective antigen.

**Table 2:** Therapeutic antibodies for viral respiratory infections.

Indication.	Generic Name	Sponsoring Company	Target	Development Stage	NCT	Completion Date	Status
*Respiratory syncytial virus*	MEDI-8897 (nirsevimab)	MedImmune	F-protein	Phase 2/3	NCT03959488, NCT03979313	2021–2023	Recruiting
	MK-1654	Merck	F-protein	Phase 2	NCT03524118	2022	Active
*Influenza A virus*	VIR-2482	Vir Biotechnology	HA	Phase 2	NCT04033406	2022	Recruiting
	CT-P27 (antibody mixture)	Celltrion	HA	Phase 2	NCT03511066	2018	Active on Celltrion website
	VIS-410	Visterra	HA	Phase 2	NCT03040141, NCT02989194, NCT02468115	2018	Active on Visterra website
*HPV-associated* *Recurrent Respiratory* *Papilloma*	Avelumab	Merck	PD-L1	Phase 2	NCT02859454	2019	Active
	Pembrolizumab	Merck	PD-1	Phase 2	NCT02632344	2020	Active, not yet recruiting

**Table 3:** Therapeutic antibodies for the COVID-19 pandemic.

Strategy	Generic Name	Sponsoring Company	Target	Development Stage	NCT (Status)	Completion Date
*ARDS-related target*	IC14	Implicit Bioscience	CD14	Phase 2	NCT04391309 (Active, not recruiting)	2021
	Itolizumab	Biocon	CD6	Phase 2	NCT04475588 (Completed)	2020
	Leronlimab	CytoDyn	CCR5	Phase 2	NCT04343651 (Active, not recruiting), NCT04347239 (Recruiting)	2021
	CPI-006	Corvus Pharmaceuticals	CD73	Phase 1	NCT04464395 (Recruiting)	2021
	Meplazumab	Jiangsu Pacific Meinuoke Bio Pharmaceutical	CD147	Phase 1	NCT04369586 (Recruiting)	2020
				Phase 1/2	NCT04275245 (Recruiting)	2020
				Phase 2/3	NCT04586153 (Not yet recruiting)	2021
	Lanadelumab	Takeda	Kallikrein	Phase 1/2	NCT04422509 (Recruiting)	2021
				Phase 1	NCT04460105 (Withdrawn)	2020
	Otilimab	GlaxoSmithKline	GM-CSF	Phase 2	NCT04376684 (Recruiting)	2020
	Lenzilumab	Humanigen	GM-CSF	Phase 2	NCT04583969 (Recruiting)	2021
				Phase 3	NCT04351152 (Recruiting), NCT04534725 (Not yet recruiting)	2020
	Gimsilumab	Kinevant Sciences Roivant Sciences	GM-CSF	Phase 2	NCT04351243 (Active, not recruiting)	2020/2021
	TJ003234	I-Mab Biopharma	GM-CSF	Phase 2/3	NCT04341116 (Recruiting)	2021
	Mavrilimumab	Kiniksa pharmaceuticals	GM-CSF-R	Phase 2	NCT04397497 (Not yet recruiting), NCT04463004 (Recruiting), NCT04399980 (Active, not recruiting), NCT04492514 (Recruiting)	2020–2021
				Phase 2/3	NCT04447469 (Recruiting)	2021
	Pamrevlumab	FibroGen	CTGF	Phase 2	NCT04432298 (Recruiting)	2020/2021
	SNDX-6352 (axatilimab)	Syndax Pharmaceuticals	CSF-1R	Phase 2	NCT04415073 (Suspended)	2020
				Phase 2	NCT04344782 (Active, Not yet recruiting), NCT04275414 (Completed)	2020
	BDB-001	Staidson Biopharmaceuticals	PD-L1	Phase 2/3	NCT04449588 (Recruiting)	2021/2022
	Nivolumab	Bristol-Myers Squibb	PD1	Phase 2	NCT04413838 (Active, Not yet recruiting)	2021
				Phase 2	NCT04343144 (Active, Not yet recruiting)	2020
				Phase 2	NCT04356508 (Not yet recruiting)	2021
				Phase 2	NCT04356508S	2020/2021
	Crizanlizumab	Novartis	P-selectin	Phase 2	NCT04435184 (Recruiting) NCT03474965 (Recruiting)	2020 2024
	CSL312 (garadacimab)	CSL Behring	Factor XIIa	Phase 2	NCT04409509 (Recruiting)	2020
	LY3127804	Eli Lilly	Angiopoietin 2	Phase 2	NCT04342897 (Terminated)	2020
	Ravulizumab	Alexion pharmaceuticals	C5	Phase 3	NCT04369469 (Recruiting) NCT04570397 (Not yet recruiting)	2020/2021
				Phase 4	NCT04390464 (Recruiting)	2021/2022
	Secukinumab	Novartis	IL-17A	Phase 2	NCT04403243 (Recruiting)	2020
	Canakinumab	Novartis	IL-1ß	Phase 2	NCT04365153 (Active, Not yet recruiting)	2020
				Phase 3	NCT04362813 (Active, not recruiting) NCT04510493 (Recruiting)	2020 2023
	Clazakizumab	Vitaeris	IL-6	Phase 2	NCT04381052 (Not yet recruiting), NCT04343989 (Recruiting), NCT04363502 (Recruiting), NCT04494724 (Recruiting), NCT04348500 (Not yet recruiting) NCT04659772 (Enrolling by invitation)	2020/2021
					NCT04348500 (Active, not recruiting)	2021
	Sirukumab	Janssen	IL-6	Phase 2	NCT04380961 (Recruiting)	2021
	Sarilumab	Regeneron Pharmaceuticals	IL-6	Phase 1	NCT04386239 (Active, Not yet recruiting)	2020
				Phase 2	NCT04357860 (Not yet recruiting), NCT04357808 (Active, not recruiting), NCT04359901 (Recruiting), NCT04661527 (Recruiting)	2020/2023
				Phase 2/3	NCT04315298 (Completed), NCT04324073 (Active, not recruiting), NCT04341870 (Suspended)	2021
				Phase 3	NCT04327388 (Completed), NCT04345289 (Recruiting)	2020/2021
	Olokizumab	R-Pharm International	IL-6	Phase 2/3	NCT04380519 (Completed), NCT04452474 (Not yet recruiting)	2020–2021
	Tozilizumab + Sarilumab	Roche, Sanofi	IL-6	Phase 2	NCT04322773 (Terminated)	2021
	Siltuximab	Janssen	IL-6	Phase 2	NCT04329650 (Recruiting)	2020
	Tocilizumab	Roche	IL-6R	Phase 1	NCT04560205 (Recruiting)	2020
				Phase 2	NCT04445272 (Recruiting), NCT04479358 (Recruiting), NCT04412291 (Recruiting), NCT04331795 (Completed), NCT04377659 (Active, not recruiting), NCT04363736 (Completed), NCT04435717 (Recruiting), NCT04377503 (Active, Not yet recruiting), NCT04339712 (Recruiting), NCT04370834 (Suspended), NCT04315480 (Active, not recruiting), NCT04331808 (Active, not recruiting), NCT04317092 (Active, Not yet recruiting) NCT04363853 (Active, Not yet recruiting)	2020–2022
				Phase 2/3	NCT04381936	2021/2031
				Phase 3	NCT04403685 (Terminated), NCT04423042 (Not yet recruiting), NCT04412772 (Recruiting), NCT04345445 (Not yet recruiting), NCT04424056 (Not yet recruiting), NCT04335071 (Terminated), NCT04361032 (Not yet recruiting), NCT04320615 (Completed), NCT04372186 (Active, not recruiting), NCT04356937 (Active, not recruiting), NCT04577534 (Recruiting)	2020–2022
				Phase 4	NCT04377750 (Recruiting)	2020/2021
	Levilimab	Biocad	IL-6R	Phase 3	NCT04397562 (Completed)	2021
	Tocilizumab + Dexamethasone	Roche	IL-6R	Phase 2	NCT04476979 (Recruiting)	2021
	Tocilizumab + Remdesivir	Roche	IL-6R	Phase 3	NCT04409262 (Recruiting)	2020
	Tocilizumab + Favipiravir	Roche	IL-6R	NA	NCT04310228 (Recruiting)	2020
	Tocilizumab +Siltuximab	Roche, Janssen	IL-6 + IL-6R	Phase 3	NCT04330638 (Active, not recruiting)	2020
	Tocilizumab + Pembrolizumab	Roche, Merck	IL-6R + PD-1	Phase 2	NCT04335305 (Recruiting)	2020
	Tocilizumab + Hydroxychloroquine + Azithromycin	Roche	IL-6R	Phase 2	NCT04332094 (Recruiting)	2020
	Heparin + Tocilizumab	Roche	IL-6R	Phase 3	NCT04600141 (Not yet recruiting)	2021
	BMS-986253	Bristol-Myers Squibb	IL-8	Phase 2	NCT04347226 (Recruiting)	2021/2022
	Risankizumab	Jiangsu Pacific Meinuoke Bio Pharmaceutical	IL-23	Phase 2	NCT04583956 (Recruiting)	2021
				Phase 2/3	NCT04586153 (Not yet recruiting)	2021
	MSTT1041A (astegolimab)	Genetech	IL-33	Phase 2	NCT04386616 (Recruiting)	2020
	Emapalumab	Swedish Orphan BIovitrum	IFN-γ	Phase 2/3	NCT04324021 (Terminated)	2020
	IFX1 (vilobelimab)	InflaRx GmbH	C5a	Phase2/3	NCT04333420 (Recruiting)	2021
	Infliximab	MSD	TNF-alpha	Phase 2	NCT04425538 (Recruiting)	2020
	Remdesivir + infliximab	MSD	TNF-alpha	Phase 3	NCT04593940 (Recruiting)	2021
	Eculizumab	Alexion pharmaceuticals	C5	Phase 2	NCT04346797 (Recruiting)	2020
	AK119	Akesobio	CD73	Phase 1	NCT04516564 (Recruiting)	2021
	CERC-002	Cerecor	LIGHT	Phase 2	NCT04412057 (Recruiting)	2020
	Glenzocimab	Acticor Biotech	Platelet glycoprotein VI	Phase 2	NCT04659109 (Not yet recruiting)	2021
	VIB7734	Viela Bio	ILT7	Phase 1	NCT04526912 (Recruiting)	2021
	NGM621	ngmbio	C3	Phase 1/2	NCT04582318 (Recruiting)	2021
	EB05	Edesa Biotech	TLR4	Phase 2/3	NCT04401475 (Not yet recruiting)	2021
	Avdoralimab	Innate Pharma	C5aR	Phase 2	NCT04371367 (Recruiting)	2020
*Virus-related target*	BRII-196	Brii biosciences	S protein	Phase 1	NCT04479631 (Active, not recruiting)	2021
	BRII-198		S protein	Phase 1	NCT04479644 (Active, not recruiting)	2021
	JS016	Shanghai Junshi Bioscience Co., Ltd.	S protein	Phase 1	NCT04441918 (Recruiting)	2020
	LY-CoV555, LY3819253	Eli Lilly	S protein	Phase 1	NCT04411628 (Completed), NCT04537910 (Active, not recruiting)	2020
				Phase 2	NCT04427501 (Recruiting)	2020
				Phase 2/3	NCT04518410 (Recruiting)	2021
				Phase 3	NCT04497987 (Recruiting), NCT04501978 (Active, not recruiting)	2021
				Phase 4	NCT04656691 (Not yet recruiting)	2021
	REGN-COV2 (REGN10933 + REGN10987)	Regeneron Pharmaceuticals	S protein	Phase 1	NCT04519437 (Active, not recruiting)	2021
				Phase 1/2	NCT04425629 (Recruiting), NCT04426695 (Recruiting)	2020–2021
				Phase 2	NCT04666441 (Not yet recruiting)	2021
				Phase 3	NCT04452318 (Recruiting)	2021
	SCTA01	Sinocelltech	S protein	Phase 1	NCT04483375 (Recruiting)	2021
				Phase 2/3	NCT04644185 (Not yet recruiting)	2021
	STI-2020 (COVI-AMG™)	Sorrento Therapeutics	S protein	Phase 1/2	NCT04584697 (Recruiting)	2021
	COVI-GUARD (STI-1499)	Sorrento Therapeutics	S protein	Phase 1	NCT04454398 (Recruiting)	2021
	TY027	Tychan Pte Ltd.	S protein	Phase 1	NCT04429529 (Active, not recruiting)	2020
				Phase 3	NCT04649515 (Not yet recruiting)	2021
	BGB-DXP593	BeiGene	S protein	Phase 1	NCT04532294 (Recruiting)	2021
				Phase 2	NCT04551898 (Not yet recruiting)	2021
	VIR-7831	Vir Biotechnology—GlaxoSmithKline	S protein	Phase 2/3	NCT04545060 (Recruiting)	2021
	MW33	Mabwell Bioscience	S protein	Phase 1	NCT04533048 (Active, not recruiting)	2020
				Phase 2	NCT04627584 (Not yet recruiting)	2021
	47D11	AbbVie	S protein	Phase 1	NCT04644120 (Not yet recruiting)	2021
	AZD7442 (AZD8895 + AZD1061)	AstraZeneca	S protein	Phase 1	NCT04507256 (Active, not recruiting)	2021
				Phase 3	NCT04625725 (Recruiting), NCT04625972 (Recruiting)	2022
	LY3832479	Junshi/Eli Lilly		Phase 2/3	NCT04441931 (Completed)	2020
	HFB30132A	Hifibio	S protein	Phase1	NCT04590430 (Recruiting)	2021
	BI767551 DZIF-10c	Boehringer Ingelheim		Phase 1/2	NCT04631705 (Not yet recruiting) NCT04631666 (Not yet recruiting)	2021
	HLX71 fusion protein	Hengenix biotech/Henlius	S protein	Phase1	NCT04583228 (Not yet recruiting)	2021
	HLX70	Hengenix biotech/Henlius	S protein	Phase 1/2	NCT04561076 (Not yet recruiting)	
	AMD03820	Olygo Bioservice		Phase 1/2	NCT04592549 (Recruiting)	2021
	CT-P59	Celltrion	S protein	Phase 1/2	NCT04525079 (Recruiting)	2020
				Phase 2/3	NCT04602000 (Recruiting)	2021

CCR, CC chemokine receptor; CD, cluster of differentiation; CSF1R, colony stimulating factor 1 receptor; CTGF, connective-tissue growth factor; GM-CSF, granulocyte monocyte colony stimulating factor; GM-CSF-R, granulocyte monocyte colony stimulating factor receptor; IFN-γ, interferon gamma; IL, interleukin; IL-R, interleukin receptor; PD1, programmed cell death 1; S protein, spike protein; TNF, tumor necrosis factor; VEGF, vascular endothelial growth factor; VEGFR, vascular endothelial growth factor receptor.

**Table 4:** Therapeutic antibodies for bacterial respiratory infections.

Indication	Generic Name	Sponsoring Company	Target	Development Stage	NCT	Completion Date	Status
*Staphylococcus aureus*	MEDI4893	AstraZeneca	α-toxin	Phase 2	NCT02296320	2018	Active on AZ pipeline
	AR-301	Aridis Pharmaceuticals	α-toxin	Phase 3	NCT03816956	2020	Active on Aridis pipeline
*Bacillus* *anthracis*	Obiltoxaximab (anthim)	Elusys Therapeutics	PA	Phase 4	NCT03088111	2020	
*Pseudomonas* *aeruginosa*	AR-101 (aerumab)	Aridis Pharmaceuticals	LPS	Phase 2	NCT00851435	2009	Active on Aridis pipeline
	AR-105 (aerucin)	Aridis Pharmaceuticals	Alginate	Phase2	NCT03027609	2019	Active on Aridis pipeline

LPS, lipopolysaccharide; PA, protective antigen.

**Table 5:** Monoclonal antibodies for fungal respiratory infections.

Indication	Generic Name	Sponsoring Company	Target	Development Stage	NCT	Completion Date	Status
*Aspergillus fumigatus*	Benralizumab	MedImmune	IL-5Rα	Phase 4	NCT04108962	2022	Recruiting
	Dupilumab	Regeneron Pharmaceuticals	IL-4/IL-13R	Phase 3	NCT04442269	2023	Active, not yet recruiting

IL, interleukin; IL-R: interleukinreceptor.

## Data Availability

Not applicable.
